# Interpreting changes in measles genotype: the contribution of chance, migration and vaccine coverage

**DOI:** 10.1186/1471-2334-8-44

**Published:** 2008-04-11

**Authors:** Shuko Nojiri, Emilia Vynnycky, Nigel Gay

**Affiliations:** 1 Department of Pharmacoepidemiology, Faculty of Medicine, University of Tokyo, 7-3-1 Hongo, Bunkyo-ku, Tokyo 113-8655, Japan; 2 Modelling and Economics Unit, Health Protection Agency Centre for Infections, 61 Colindale Avenue, London, NW9 5EQ, UK

## Abstract

**Background:**

In some populations, complete shifts in the genotype of the strain of measles circulating in the population have been observed, with given genotypes being replaced by new genotypes. Studies have postulated that such shifts may be attributable to differences between the fitness of the new and the old genotypes.

**Methods:**

We developed a stochastic model of the transmission dynamics of measles, simulating the effects of different levels of migration, vaccination coverage and importation of new genotypes on patterns in the persistence and replacement of indigenous genotypes.

**Results:**

The analyses illustrate that complete replacement in the genotype of the strain circulating in populations may occur because of chance. This occurred in >50% of model simulations, for levels of vaccination coverage and numbers of imported cases per year which are compatible with those observed in several Western European populations (>80% and >3 per million per year respectively) and for the given assumptions in the model.

**Conclusion:**

The interpretation of genotypic data, which are increasingly being collected in surveillance programmes, needs to take account of the underlying vaccination coverage and the level of the importation rate of measles cases into the population.

## Background

Genotyping is increasingly being used in measles surveillance programmes and to monitor the success of elimination programmes [1]. In some populations, e.g. Germany, complete shifts in the genotype of the predominant circulating strain have been observed, with given genotypes being replaced altogether by new genotypes [2].

Such changes have been attributed to an increased fitness of the new genotype, as compared with that of the previous genotype circulating in the population [3]. In practice, such changes may also occur because of many different factors, including chance, the level of control achieved through vaccination and the number of measles viruses which enter the population through importations. For example, in populations in which many individuals have been immunized, the diversity of strains isolated from cases can be high, since most cases may be linked to importations from other areas, and the high level of immunity in the population results in most of the chains of transmission being small. In such situations, only a few of the imported strains can establish themselves, with the probability of this occurring depending on the importation rate.

To date, the circumstances under which replacement and persistence of genotypes in a population occurs is poorly understood. Using a stochastic model of the transmission dynamics of measles, we quantify the levels of vaccination coverage and the rate at which measles viruses enter the population which are associated with different patterns in persistence and replacement of existing genotypes circulating in the population.

## Methods

### General overview of the model

The model simulates the introduction and transmission of measles strains with new genotypes into a population in which, in the absence of importations, a single dominant measles genotype is circulating. In the first instance, we consider a population comprising one city with 500,000 individuals and with regular importations of a new genotype. We also consider an extension of this model, namely a population comprising two cities, each with 500,000 individuals and different degrees of interaction between the two cities. The latter scenario is comparable to the situation which might be seen e.g. in a country.

### Description of the model

The population in each city is stratified into individuals who are susceptible to measles infection, those who are infectious and those who are immune, either because of previous infection or vaccination. For simplicity, individuals are not stratified by age. Vaccination or previous infection with a strain with a given genotype is assumed to provide solid lifelong protection against infection with all genotypes. The model formulation is based on the classic (discrete generation) Hamer approach, which was subsequently used by Fine and Clarkson to analyse seasonality in contact patterns[4,5]. The equations used to formulate the model are provided in the Additional file 1.

Contact between individuals in a given city is assumed to be random. In the two-city model, contact between individuals in different cities is assumed to be non-random. It is determined by a parameter e (ranging between 0.01 and 0.5), which reflects the proportion of contacts that an individual in a given city makes with individuals in the other city, with e = 0.5 corresponding to random mixing between individuals in the two cities (see Additional file 1). New genotypes were assumed to enter the population as a result of importations.

The model was designed to be stochastic, with chance determining the number of new infections with any given genotype, the number of births and deaths into or out of the population, and whether or not an imported case infected with a new measles genotype entered the population. This meant that, in contrast with a deterministic model, which describes what happens on average in a population, the model can provide estimates of the probability of replacement or persistence of an imported genotype. Table 1 summarizes the key input parameters in the model.

The model was run 500 times considering a 40 year time period, for levels of vaccination coverage of 0–90% and importation rates of 1 and 3 per million per year to determine the probability of the following patterns of replacement and persistence of given genotopes occurring:

1.1. Indigenous measles genotype persists, but cases infected with an imported genotype are observed only rarely.

1.2. Indigenous measles genotype persists, while cases infected with an imported genotype are observed, but the transmission of this genotype does not persist.

2. Both indigenous measles genotype and imported genotype persist during the study period.

3.1. Indigenous measles genotype dies out, while the imported genotype gradually becomes dominant.

3.2. Indigenous measles genotype dies out, while the imported genotype becomes dominant quickly.

4. Transmission of neither genotype persists throughout the study period.

**Figure 1:**
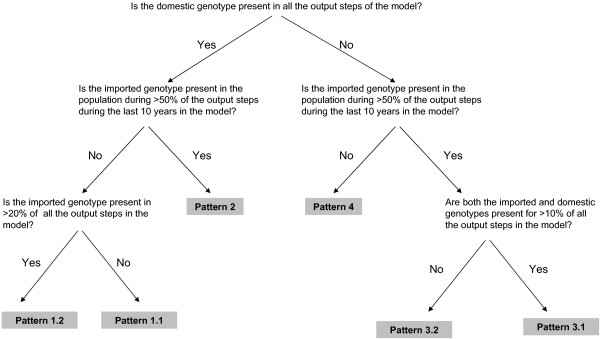
Summary of the criteria used to define the various patterns in the persistence and replacement of measles genotypes.

To our knowledge, patterns in the persistence of genotypes have not been categorized to date, though the above categorization appears reasonable. The probability of a given pattern in persistence and replacement occurring was calculated as the proportion of simulations for which it occurred for the given vaccination and importation assumption. The range in the importation rates used are compatible with those estimated for England and Wales[6]. Figure 1 summarizes the criteria used to define the occurrence of each of the above patterns. In particular, the criteria used to define pattern 4 (transmission of neither genotype persists throughout the study period) differs from the definition of fade out which has been used to estimate the critical population size for measles as being ~300,000. Pattern 4 is here defined as the presence of the domestic genotype in <100% of all the output steps, and the presence of the imported genotype in <50% of the output steps during the final 10 years of the model simulations – see Figure 1). In contrast, past analyses have defined fade out as the absence of cases over a period of at least 3 weeks [7-9].

**Figure 2:**
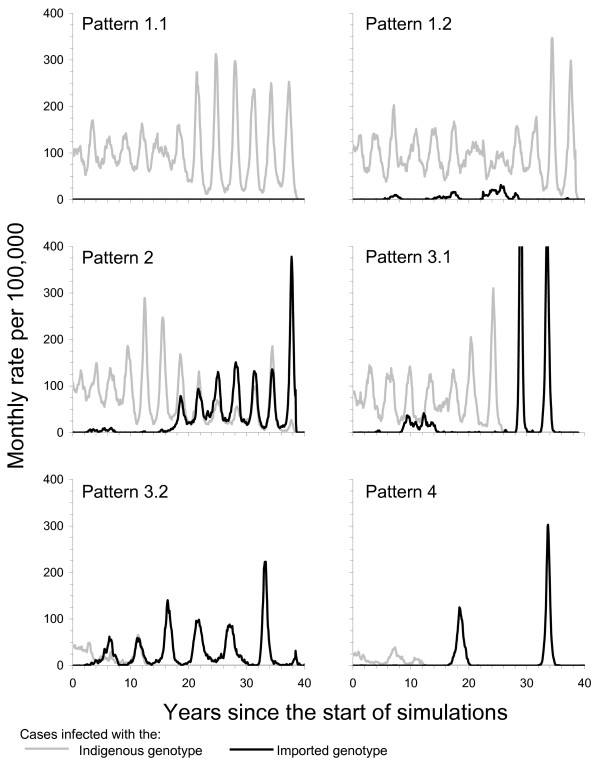
**Summary of the definitions of the patterns in the persistence and replacement of the indigenous and imported measles genotypes used in the model.** See Figure 1, the Methods and Additional file 1 for further details.

Figure 2 summarizes the trends in the incidence of cases infected with the indigenous and imported genotype corresponding to these patterns. Further details of the calculations are provided in the Additional file 1.

## Results

**Figure 3:**
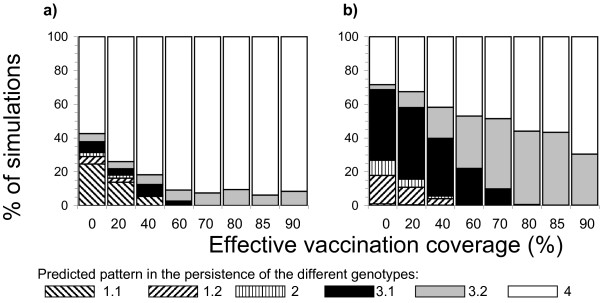
Summary of the proportion of simulations of measles transmission in a single city, in which the various patterns in the persistence and replacement of the indigenous and imported genotypes were seen, assuming that the importation rate was a) 1 case/million/year and b) 3 cases/million/year, for different levels of effective vaccination coverage.

Figure 3a summarizes the proportion of simulations of measles transmission in a single city in which the different patterns in genotype persistence and replacement occurred, for different levels of vaccination coverage and assuming a low importation rate (1/million/year).

At low levels of vaccination coverage, all patterns in persistence and replacement were predicted to occur. However, pattern 4 (transmission of neither genotype persists during the study period) and pattern 1.1 (indigenous genotype persists but cases infected with the imported genotype are observed only rarely) were the most likely, occurring in 55% and 25% of simulations respectively if no individuals were vaccinated. The probability of pattern 4 occurring increased as the vaccine coverage increased, occurring in 90% of the simulations once the vaccination coverage was ≥60%, with rapid replacement of the indigenous genotype occurring in the remaining (10%) simulations.

With a high importation rate (3/million/year – see Figure 3b), the distribution of patterns in persistence and replacement changed. With no vaccination, gradual replacement with the indigenous genotype (pattern 3.1) was the pattern which was most likely to occur, accounting for 50% of the simulations. Its relative importance decreased as the vaccination coverage increased, whilst the proportion of simulations in which rapid replacement of the existing genotype occurred increased, reaching 30–45% under high levels of vaccination coverage.

**Figure 4:**
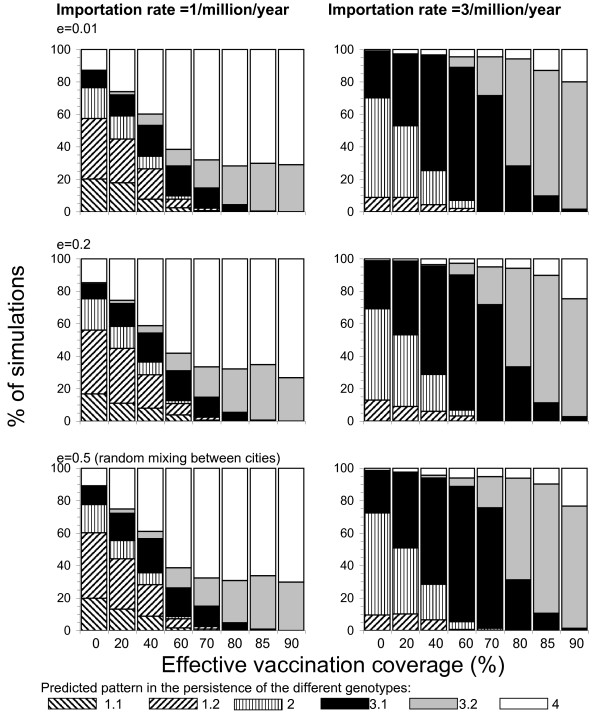
Summary of the proportion of simulations in which the various patterns in the persistence and replacement of the indigenous and imported genotypes were seen in the two city model, assuming that the importation rate was either 1 or 3 cases/million/year, for different levels of effective vaccination coverage and for different assumptions about mixing between individuals in the model (i.e. e ranging between 0.01 and 0.5, corresponding to minimal and random mixing respectively between individuals in the two cities).

The corresponding predictions for a population comprising two cities, with different degrees of interaction between them, are summarized in Figure 4. In general, for a given importation rate, the predictions were comparatively insensitive to assumptions about contact between cities. For both assumptions about the importation rate, rapid replacement of the indigenous genotype was unlikely to occur if the vaccination coverage was low e.g. occurring in 0–2% of the simulations if no individuals were vaccinated. However, the probability of rapid replacement occurring increased as the vaccination coverage increased, accounting for 30% and 70% of the simulations if the importation rate was 1 and 3 per million per year respectively, and if the vaccination coverage was ≥80%.

## Discussion

Genotyping is increasingly being applied in the surveillance of measles transmission. To date, several patterns in the persistence and replacement of measles strains have been observed. In Germany, a rapid reduction in the prevalence of indigenous genotypes, including C2 and D6, has been seen, which has coincided with the emergence of D7 viruses[2]. It has been suggested that such changes may occur because of an increased fitness of D7 genotypes as compared with that of the C2 and D6[3]. Our analyses indicate that such changes can occur by chance in most settings, irrespective of the fitness of the genotypes and need not have negative implications for the success of elimination programmes.

For example, in settings with a low vaccination coverage, model predictions suggested that all patterns in the persistence and replacement could occur. In settings with a high vaccination coverage e.g. >80%, rapid replacement of the indigenous genotype was predicted to occur in 30% of the model simulations, even if the importation rate was low (1 case per million per year – see Figure 4). In settings in which the importation rate of new measles genotypes was high e.g. 3 cases per million per year, rapid replacement of the indigenous genotype was predicted to occur in approximately 70% of the simulations if the vaccination coverage was over 80% (Figure 4).

The levels of vaccination coverage associated with rapid replacement in the model are compatible with those which have been observed in Germany and other European populations. Since 1990, for example, trivalent measles-mumps-rubella (MMR) vaccine has been recommended in Germany for those aged 11–14 months (1st dose) and 15–23 months (2nd dose). Vaccination coverage during the period 1998–2001 for the first dose was reported to be 90.3% and 20% for the second dose [10]. Similarly high vaccination coverages of over 80% have been observed elsewhere in Europe, e.g. France, Denmark, Finland, the UK and Sweden [11], though the patterns in the persistence and replacement in measles strains which have occurred in these settings remain unknown.

We have considered two types of settings in our analyses, namely a population comprising a single city (which is analogous to the situation e.g. in an island) and a population comprising 2 cities, each comprising 500,000 individuals with differing degrees of interaction between the two. Many different, and potentially, more realistic, assumptions relating to population structure or contact between individuals could have been incorporated in the model e.g. considering populations comprising five or ten different cities and where contact between individuals depends on age and social group. Such assumptions probably did not influence our conclusions substantially, given that rapid replacement was predicted to occur for all assumptions about contact between individuals in the model population, if the vaccination coverage was in a realistic range (i.e. ≥40%) and if the importation rate was about 3 cases per million per year. Such importation rates are compatible those observed in reality [6].

The model has examined the situation in which only 2 genotypes are circulating in a population and all imported cases are infected with same genotype. It is recognized that, in reality, the diversity of genotypes circulating in a population is strongly related to the extent of control of measles transmission and the genotypes observed are sometimes specific to the setting.

In central and western Africa for example, members of clade B have been isolated, whereas the A, D2 and D4 genotypes have been found in South Africa [12]. In populations in which control of measles transmission has not been achieved, the diversity is typically low [13]. In China [14], Nepal and Japan [15], one or two distinct genotypes have been observed. In some African settings, a few domestic MV strains have remained endemic e.g. in Nigeria and Ghana, two distinct virus genotypes appear to be co-circulating [16]. In Russia and central Europe, genotype intermixing occurs, with a number of distinct genotypes having been seen in several countries during the late 1980s and 1990s [3]. In contrast, in the USA and Germany, where vaccination coverage has been high, several different genotypes have been observed. Phylogenetic analysis in Germany revealed the presence of at least six measles virus genotypes including B3, C2, D4, D6, G2 and D7.

Recent workers have suggested that certain MV with a lower susceptibility to existing neutralizing antibodies may have a selective advantage for replacing the domestic MV genotype [2] and that the wild type MVs have various features which means that they react with different monoclonal antibodies and lead to nucleotide sequence diversity. To date, there has been little discussion of whether the D7 genotype has any selective biological advantage. Though this is plausible, the fact that the D7 genotype does not yet predominate in other countries suggests that this may be unlikely.

Our analyses have not accounted for potential genetic or antigenic drift in the measles virus. So far, any mutational drifts, such as those which have been observed in South Africa between the late 1980s and the mid 1990s[12] or in Madrid between the late 1960s and mid 1990s [17], have been small. As a result, vaccinated individuals have typically been protected against infection with these drift variants. It is biologically plausible that significant genetic drift in RNA viruses due to lack of proof reading by RNA polymerase may result in a lowering of the vaccine efficacy. For example, Fayolle et al characterized a natural mutation in an antigenic site involved in neutralization and showed antigenic drift by vaccine antibodies [18].

Some of the issues discussed may well be relevant for interpreting molecular epidemiological data for mumps, rubella and other infections. Based on the results derived from encoding five protein products, the E1 gene sequence has been used for genotyping and phylogenetic analyses of rubella viruses [19]. Rubella viruses from Europe, Asia, and North America have belonged to a single genotype (Rubella Genotype I or RGI), while in Asia (China and India) and Italy, a distant phylogenetic branch was found, which has been designated Rubella Genotype II (RGII). Du-Ping Zheng et al indicated that the temporal pattern of isolation of viruses in the European branches and sub-branches might be related to temporal displacement of genotypic groups[19]. Inou Y el al have also investigated the distribution of mumps virus genotypes in Japan by nucleotide sequencing part of the hemagglutinin-neuraminidase (HN) and small hydrophobic (SH) protein regions and have found that genotypes B and K co-circulated in the 1990s and had been replaced by genotype G in the year 2000 [20].

A recent review of the global distribution of measles genotypes has highlighted the fact that the distribution, diversity and types of measles genotypes differs substantially between populations [21]. Overall, our analyses have highlighted that the patterns in the persistence and replacement of measles genotypes depend on several factors, including the vaccination coverage and the number of cases imported into the population each year. In addition they have suggested that complete replacement measles of genotypes, such as that which has been observed in Germany [3], could occur by chance, with the probability being greatest if both the vaccination coverage and the number of cases imported into the population are high. Such insights should prove useful for interpreting patterns in the persistence and replacement of genotypes which may occur in the future.

## Conclusion

In this study, we illustrate that sudden genotype shifts may occur as a result of chance, with their probability of occurrence being about 70% for levels of vaccination coverage and numbers of imported cases per year which are compatible with those observed in several Western European populations (>80% and >3 per million per year respectively), using a stochastic model of the transmission dynamics of measles. These findings suggest that the interpretation of genotypic data needs to take account of the underlying vaccination coverage and the level of the importation rate of measles cases into the population.

## Competing interests

The author(s) declare that they have no competing interests.

## Authors' contributions

SN set up the model, carried out the analyses and wrote the manuscript. EV contributed to the implementation of the model and the analyses and helped write the manuscript. NG conceived of the study, participated in its design and co-ordination and contributed in writing the manuscript. All the authors have read and approved the final manuscript.

## Pre-publication history

The pre-publication history for this paper can be accessed here:



## Supplementary Material

Additional file 1 Technical details of the modeling methods. The file provides further technical details of the modelling methods used in the manuscript. Click here for file
