# Supplementary material for: Interpreting changes in measles genotype: the contribution of chance, migration and vaccine coverage
Source: BMC Infect Dis. 2008 Apr 11;8:44. doi: 10.1186/1471-2334-8-44 (PMC2346460; doi:10.1186/1471-2334-8-44)
Supplement: Additional file 1 — Technical details of the modeling methods. The file provides further technical details of the modelling methods used in the manuscript. [file 1471-2334-8-44-S1.pdf]

Technical details of the modelling methods

General description of the model

The population in a given city is structured into individuals who are susceptible, infectious and immune (recovered) and, for simplicity, the population is not stratified by age. Once immune (either through vaccination or following natural infection), individuals are assumed to remain immune for life, and vaccination is assumed to protect equally against all genotypes.

At the start of the simulations, infectious individuals in the population are infected with a single genotype (genotype "i"), and imported cases are infected with a genotype which differs from that circulating in the original population (genotype "j"). The infection was assumed to be at endemic equilibrium at the start of simulations (t=0), with the number of susceptible individuals in a given city equaling the ratio between the population size and the R_0 in that city (population size in that city/R_0), and the number of cases equaling the number of unvaccinated individuals born into the city in each time step. Tables 1 and 2 summarize the definitions of the variables and parameters used in the model. The equations describing the model formulation for a given city are summarized below, where, for notational convenience, the superscripts used for referring to specific cities have been dropped:

$$S_{t+1} = S_t - (n_t^i + n_t^j) + b_t(1-v) - d_t^S \qquad (1)$$
$$I_{t+1} = I_t + n_t^i - d_t^I - r_t^I \qquad (2)$$
$$J_{t+1} = J_t + n_t^j - d_t^J - r_t^J + y_t^j \qquad (3)$$
$$R_{t+1} = R_t + b_t v + r_t^I + r_t^J - d_t^R \qquad (4)$$

The equations were set up using time steps of 1 serial interval (approximately 14 days) using a specially-written C program, with the simulations spanning a period of 38 years (1,000 serial intervals). The model findings were also checked using the same model set up in Excel. Chance was assumed to determine the number of new infections, births into the population, and the numbers of deaths from each of the compartments in each time step as described below.

The number of individuals in a given category (e.g. susceptibles, infectious, immune) who died in a given city z in each time step was calculated by drawing a random number from the Binomial distribution, with parameters consisting of the number of individuals in that compartment at time t (e.g. S_t^z) and the death rate. The number of individuals born into a given city z during each time step was calculated in an analogous way i.e. by drawing a random number from the Binomial distribution, with parameters N_t^z and the birth rate. The number of individuals newly infected with genotype k (=i or j) between t and t+1 in a given city z (=a or b) was calculated by drawing a random number from the Binomial distribution with parameters S_t^z and the risk of infection between time t and t+1 for that genotype for that city ($\lambda_t^{z,k}$). For cities a and b, this risk of infection with genotype i was calculated using the following expressions:

$$\lambda_t^{a,i} = \frac{I_t^a R_0(1-e) + I_t^b R_0 e}{N_t^a}$$

$$\lambda_t^{b,i} = \frac{I_t^a R_0 e + I_t^b R_0(1-e)}{N_t^b}$$

The corresponding expressions considering genotype j are analogous.

The entry of one imported case during each time step was determined by drawing a random number: if the random number was less than the importation rate, then a case was assumed to enter the population during that time step; otherwise no case was imported into the population during that time step.

Calculating the probability of observing different patterns in the persistence of the indigenous and imported genotype

The criteria used to define the occurrence of the various patterns in the persistence of the indigenous and imported genotypes (see the main text) are summarized in Figure 1. Variables were set up to calculate the proportion of time steps in the simulations during

which a given genotype was predicted to be circulating in the population. The probability of observing a given pattern in the persistence of the indigenous and imported genotypes for different assumptions about the vaccination coverage, importation rates and contact between cities was then calculated by repeating the simulations 500 times and calculating the proportion of simulations for which that particular pattern, as defined in Figure 1, occurred.

Table 1: Summary of the definitions of parameters used in the model

Symbol	Definition and assumption
e	Proportion of contacts made by an individual in a given city which are with individuals in the other city. e was assumed to range between 0.01 (minimal mixing between the individuals in the two cities) and 0.5 (random mixing between individuals in the two cities).
b	Birth rate. Assumed to equal the mortality rate (to ensure that the population size remained roughly constant over time).
m	Mortality rate. Assumed to equal, as by convention, 1/life expectancy, where the life expectancy is 70 years.
p	Number of importations per year into a given city, assumed to be 1 or 3/million/year. All imported cases are assumed to be infected with a strain which differs from the indigenous strain circulating in the population.
v	Effective vaccination coverage among newborns – assumed to range between 0 and 90%.
R_0	Basic reproduction number – assumed to equal 13 and to be identical for both the indigenous and imported genotype.

Table 2: Summary of the definitions of variables used in the model

Symbol	Definition and assumption
S_t^z	Number of susceptible individuals in city z at time t.
I_t^z	Number of infectious individuals in city z at time t who are infected with genotype i.
J_t^z	Number of infectious individuals in city z at time t who are infected with genotype j.
R_t^z	Number of immune individuals in city z at time t.
N_t^z	Population size of city z at time t. At the start of the simulations, 500,000 individuals were assumed to be present in each city.
$n_t^{z,k}$	Number of individuals in city z who have been newly infected with genotype k (=i or j) between time t and t+1. $n_t^{z,k}$ is assumed to follow the Binomial distribution with parameters S_t^z and $\lambda_t^{z,k}$.
$\lambda_t^{z,k}$	Risk of infection with genotype k (=i or j) in city z between time t and t+1.
$d_t^{z,S}$	Number of susceptible individuals in a given city who die between time t and t+1. $d_t^{z,S}$ is assumed to follow the Binomial distribution with parameters S_t^z and m.
$d_t^{z,I}, d_t^{z,J}$	Number of infectious individuals (infected with genotypes I and J respectively) in city z who die between time t and t+1. $d_t^{z,I}$ is assumed to follow the Binomial distribution with parameters I_t^z and m; $d_t^{z,J}$ is assumed to follow the Binomial distribution with parameters J_t^z and m.
$d_t^{z,R}$	Number of immune individuals in a given city z who die between time t and t+1. $d_t^{z,R}$ is assumed to follow the Binomial distribution with parameters R_t^z and m.
y_t^z	Number of infectious individuals who enter city z between time t and t+1. y_t^z =1 if a random number drawn is less than the importation rate, p, into that city and 0 otherwise.
b_t^z	Number of individuals who are born into a given city z between time t and t+1. b_t^z is assumed to follow the Binomial distribution with parameters N_t^z and the birth rate (which equals the mortality rate m).
$r_t^{z,I}, r_t^{z,J}$	Number of infectious individuals (infected with genotypes I and J) in a given city z who recover and become immune between time t and t+1. All infectious individuals are assumed to recover during the course of 1 serial interval (the time step used in the model), unless they die in the meantime.
